# Activation studies of the β-carbonic anhydrases from *Escherichia coli* with amino acids and amines

**DOI:** 10.1080/14756366.2020.1781845

**Published:** 2020-06-24

**Authors:** Alessio Nocentini, Sonia Del Prete, Margaret D. Mastrolorenzo, William A. Donald, Clemente Capasso, Claudiu T. Supuran

**Affiliations:** a Università degli Studi di Firenze, Dipartimento Neurofarba, Sezione di Scienze Farmaceutiche e Nutraceutiche, Sesto Fiorentino, Italy;; b Department of Biology, Agriculture and Food sciences, CNR, Institute of Biosciences and Bioresources, CNR, Napoli, Italy; c San Diego (UCSD), University of California, San Diego, CA, USA; d School of Chemistry, University of New South Wales, Sydney, Australia

**Keywords:** *E. coli*, carbonic anhydrase, activator, amino acid, enzyme kinetics

## Abstract

A β-carbonic anhydrase (CA, EC 4.2.1.1) from the widespread bacterium *Escherichia coli* (EcoCAβ), encoded by the CynT2 gene, has been investigated for its catalytic properties and enzymatic activation by a panel of amino acids and amines. EcoCAβ showed a significant catalytic activity for the hydration of CO_2_ to bicarbonate and a proton, with a kinetic constant *k*_cat_ of 5.3 × 10^5^ s^−^ and a Michaelis–Menten constant *K*_M_ of 12.9 mM. The most effective EcoCAβ activators were L- and D-DOPA, L-Tyr, 4-amino-Phe, serotonin and L-adrenaline, with *K*_A_s from 2.76 to 10.7 µM. L-His, 2-pyridyl-methylamine, L-Asn and L-Gln were relatively weak activators (K_A_s from 36.0 to 49.5 µM). D-His, L- and D-Phe, L- and D-Trp, D-Tyr, histamine, dopamine, 2-(aminoethyl)pyridine/piperazine/morpholine, L-Asp, L- and D-Glu have *K*_A_s from 11.3 to 23.7 µM. Endogenous CA activators may play a role in bacterial virulence and colonisation of the host.

## 1. Introduction

The presence of carbonic anhydrase (CA, EC 4.2.1.1) in *Escherichia coli* was first reported in the early 90s by Guilloton et al.^1^ The cyn operon present in the genome of this widespread bacterium encodes for at least three such enzymes all belonging to the β-CA genetic family, two with CO_2_ hydrase activity and one reported to act as a cyanase, i.e., catalysing the reaction of cyanate with bicarbonate to give ammonia and CO_2_^1^. One of these β-CAs that is encoded by the CynT2 gene was crystallised by Cronk et al.^2^^,^^3^ which was one of the first representatives of this class of enzymes to be structurally characterised in detail. In the same study, the authors also qualitatively measured the CO_2_ hydrase activity for this enzyme, demonstrating the enzyme to be active at a pH 8.4, but devoid of any activity at lower, or even neutral pH values. However, the detailed kinetic/thermodynamic parameters or the catalytic efficacy of this enzyme has not reported in that^2^ or any other studies to date.

Why are CAs important for *E. coli*^4^, or more generally for bacteria^5^? These enzymes are present in most organisms investigated to date^5–9^, with eight genetically distinct classes of CAs having been reported to date, the α-, β-, γ-, δ-, ζ-, η-, θ- and ι-classes^9–12^. They all catalyse the simple but crucial interconversion reaction between CO_2_ and bicarbonate, with the concomitant generation of hydronium ions:

$${\text{CO}}_{2}+{\;2H}_{2}O⇌{\text{ HCO}}_{3}^{-}+{\text{ H}}_{3}O^{+}$$

In addition to being involved in pH regulation in all organism and tissues in which they are present^13^^,^^14^, CAs are also metabolic enzymes^15^. Indeed, essential metabolic processes require either CO_2_ or bicarbonate as substrates for carboxylating reactions. Although both CO_2_ and bicarbonate spontaneously equilibrate in solution at the physiologic pH, the low concentration of CO_2_ in the air and its rapid diffusion from cells lead to an insufficient bicarbonate availability for *in vivo* metabolic and biosynthetic requirements in bacteria and other organisms^4^. For example, Merlin et al.^4^ calculated that in *E. coli*, the demand for bicarbonate is 1000–10,000-fold higher than the amount of this anion that is provided by uncatalyzed CO_2_ hydration. Thus, an enzymatic conversion of CO_2_ to bicarbonate is therefore strictly required for the growth of *E. coli* and many other bacteria^5^, which is likely why at least four CA genetic families are present in bacteria, the α-, β-, γ- and ι-CAs^5^^,^^10^^,^^12^.

In fact, inhibition of CAs belonging to various classes and organisms is exploited pharmacologically for various applications^16–22^. Many of the human isoforms (among the 15 presently known) are targets for diuretics, antiobesity, antiepileptic, antiglaucoma or antitumor agents^16–20^. Inhibition of such enzymes from pathogenic bacteria, fungi or protozoans was proposed as a new approach to develop antiinfective agents with novel mechanisms of action, devoid of the drug resistant problems of the currently used agents^21^^,^^22^. Thus, a large number of drug design studies of CA inhibitors (CAIs) targeting both mammalian and pathogenic CAs are constantly being reported^23^^,^^24^.

On the other hand, activation studies of various classes of CAs have progressed slowly compared to the inhibition studies. The CA activation mechanism was in fact explained at the molecular level only in 1997 with the report of the first X-ray crystallographic adduct of a CA-activator complex, more precisely the CA II complexed with histamine^25^. Indeed, it has been demonstrated by Briganti et al.^25^ and others^8^ that the CA activators (CAAs) participate directly in the enzyme catalytic cycle, which can be represented schematically by Equations 1 and 2.

(1) $${\text{EZn}}^{2+}-{\text{OH}}^{-}+{\text{CO}}_{2}⇌{\text{EZn}}^{2+}-{\text{HCO}}_{3}^{-}\overset{+H_{2}O}{⇌}{\text{EZn}}^{2+}-{\text{OH}}_{2}+{\text{HCO}}_{3}^{-}$$

(2) $${\text{EZn}}^{2+}-{\text{OH}}_{2}⇌{\text{EZn}}^{2+}-{\text{OH}}^{-}+H^{+}\;-\text{rate determining step}-$$

The metal hydroxide species of the enzyme (EZn^2+^-OH^−^) acts as a strong nucleophile (at physiologic pH) and converts CO_2_ to bicarbonate, which is subsequently coordinated to the catalytic metal ion [Step 1 in Equation (1)]. This adduct is not very stable and its reaction with an incoming water molecule leads to liberation of bicarbonate in solution (Step 2 in Equation (1) and generation of an acidic form of the enzyme incorporating a Zn^2+^(OH_2_) species at the metal centre, which is catalytically ineffective for the hydration of CO_2_.^6–8^^,^^25^ In order to regenerate the nucleophilic species, a proton transfer reaction occurs, which is rate determining for the catalytic cycle in many CAs [Equation (2)]. For many human isoforms, this step is assisted by a proton shuttle residue, which is His64 in most mammalian CAs. In the presence of an activator molecule ‘A’, Equation (2) becomes Equation (3); that is, in the enzyme-activator complex the proton transfer reaction is no longer intermolecular but intramolecular, and thus favoured^25–28^:

(3) $$\begin{array}{c} {\text{EZn}}^{2+}—{\text{OH}}_{2}+A⇌[{\text{EZn}}^{2+}—{\text{OH}}_{2}-\;A]\;⇌[{\text{EZn}}^{2+}—{\text{HO}}^{-}{-\;\text{AH}}^{+}]⇌\;{\text{EZn}}^{2+}—{\text{HO}}^{-}+{\;\text{AH}}^{+}\\ \text{enzyme}\;-\;\text{activator}\;\text{complexes} \end{array}$$

The imidazole moiety of the key histidine residue, with a pKa of 6.0–7.5 (depending on the isoform^6^) is an appropriate proton shuttling residue which transfers the proton from the metal coordinated water to the reaction medium, in the crucially important rate-determining step of the catalytic cycle^1–3^. The process can also be assisted by endogenous molecules, which bind within the enzyme active site, as proven by X-ray crystallography and other techniques, which have been termed CAAs^25–28^. Such activators facilitate the proton transfer reactions between the metal ion centre and the external medium by an alternative pathway to the proton shuttle residue.

**Figure 1.**
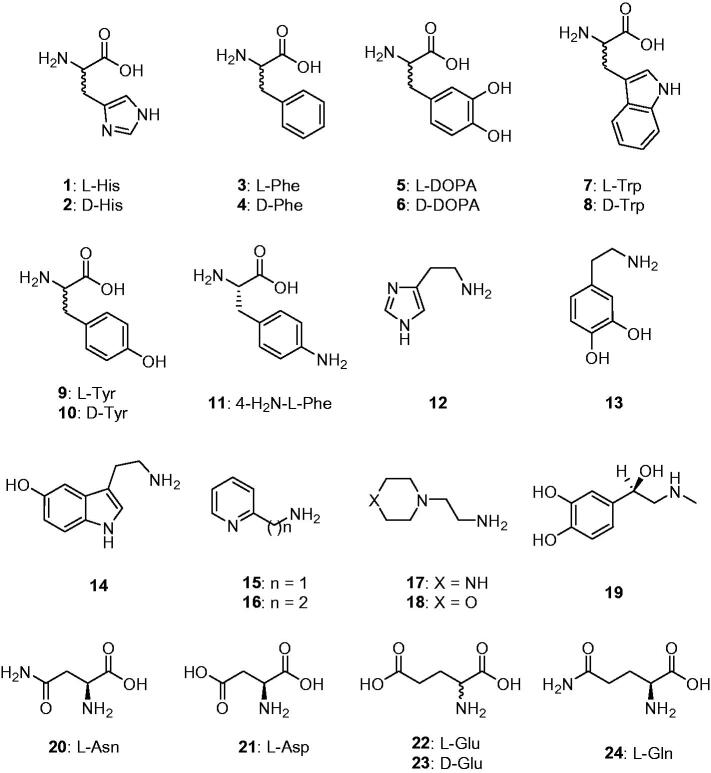
Amino acids and amines **1–24** investigated as CAAs in the current article.

CAAs were recently demonstrated to have potential pharmacologic applications, as the activation of mammalian enzymes was shown to enhance cognition and memory in experimental animals^29^, whereas its inhibition had the opposite effect^13^^,^^14^. The activation of CAs from pathogenic bacteria may also be relevant for understanding the factors governing virulence and colonisation of the host, because pH in the tissues surrounding the pathogens likely plays a key role in such processes^5^^,^^10^^,^^12^. Considering such evidence, in this study we report the first activation study with amines and amino acids (compounds **1–24**, Figure 1) of one of the β-CAs reported to be present in the model organism *E. coli*.

## 2. Materials and methods

### 2.1. Enzyme production and purification

The protocol described in Ref.^3^ has been used to obtain purified recombinant EcoCAβ.

### 2.2. Ca activity/activation measurements

An Sx.18Mv-R Applied Photophysics (Oxford, UK) stopped-flow instrument has been used to assay the catalytic activity of various CA isozymes for CO_2_ hydration reaction^30^. Phenol red (at a concentration of 0.2 mM) was used as indicator, working at the absorbance maximum of 557 nm, with 10 mM Hepes (pH 7.5, for α-CAs)^31–34^ or TRIS (pH 8.3, for β-CAs)^35–38^ as buffers, 0.1 M NaClO_4_ (for maintaining constant ionic strength), following the CA-catalyzed CO_2_ hydration reaction for a period of 10 s at 25 °C. The CO_2_ concentrations ranged from 1.7 to 17 mM for the determination of the kinetic parameters and inhibition constants. For each activator, at least six traces of the initial 5–10% of the reaction have been used for determining the initial velocity. The uncatalyzed rates were determined in the same manner and subtracted from the total observed rates. Stock solutions of activators (at 0.1 mM) were prepared in distilled-deionised water and dilutions up to 1 nM were made thereafter with the assay buffer. Enzyme and activator solutions were pre-incubated together for 15 min prior to assay, in order to allow for the formation of the enzyme–activator complexes. The activation constant (K_A_), defined similarly with the inhibition constant K_I_, can be obtained by considering the classical Michaelis–Menten equation [Equation (4)], which has been fitted by non-linear least squares by using PRISM 3:

(4) $$v\;=v_{\text{max}}/\left\{1+\left(K_{M}/\left[S\right]\right)\left(1+{\left[A\right]}_{f}/K_{A}\right)\right\}$$

where [*A*]_f_ is the free concentration of activator.

Working at substrate concentrations considerably lower than *K*_M_ ([*S*] ≪ *K*_M_), and considering that [*A*]_f_ can be represented in the form of the total concentration of the enzyme ([*E*]_t_) and activator ([*A*]_t_), the obtained competitive steady-state equation for determining the activation constant is given by Equation (5):

(5) $$v=v_{0}.K_{A}/\left\{K_{A}+{\left({\left[A\right]}_{t}-0.5\{\left({\left[A\right]}_{t}+{\left[E\right]}_{t}+K_{A}\right)-{\left({\left[A\right]}_{t}+{\left[E\right]}_{t}+K_{A}\right)}^{2}-4{\left[A\right]}_{t}.{\left[E\right]}_{t}\right)}^{1/2}\right\}\}$$

where *v*_0_ represents the initial velocity of the enzyme-catalyzed reaction in the absence of activator^35–39^. This type of approach to measuring enzyme-ligand interactions is in excellent agreement with recent results from native mass spectrometry measurements^40^.

### 2.3. Reagents

Amines and amino acid derivatives **1–24** were obtained in the highest purity that was available commercially from Sigma-Aldrich (Milan, Italy).

## 3. Results and discussion

**Figure 2.**
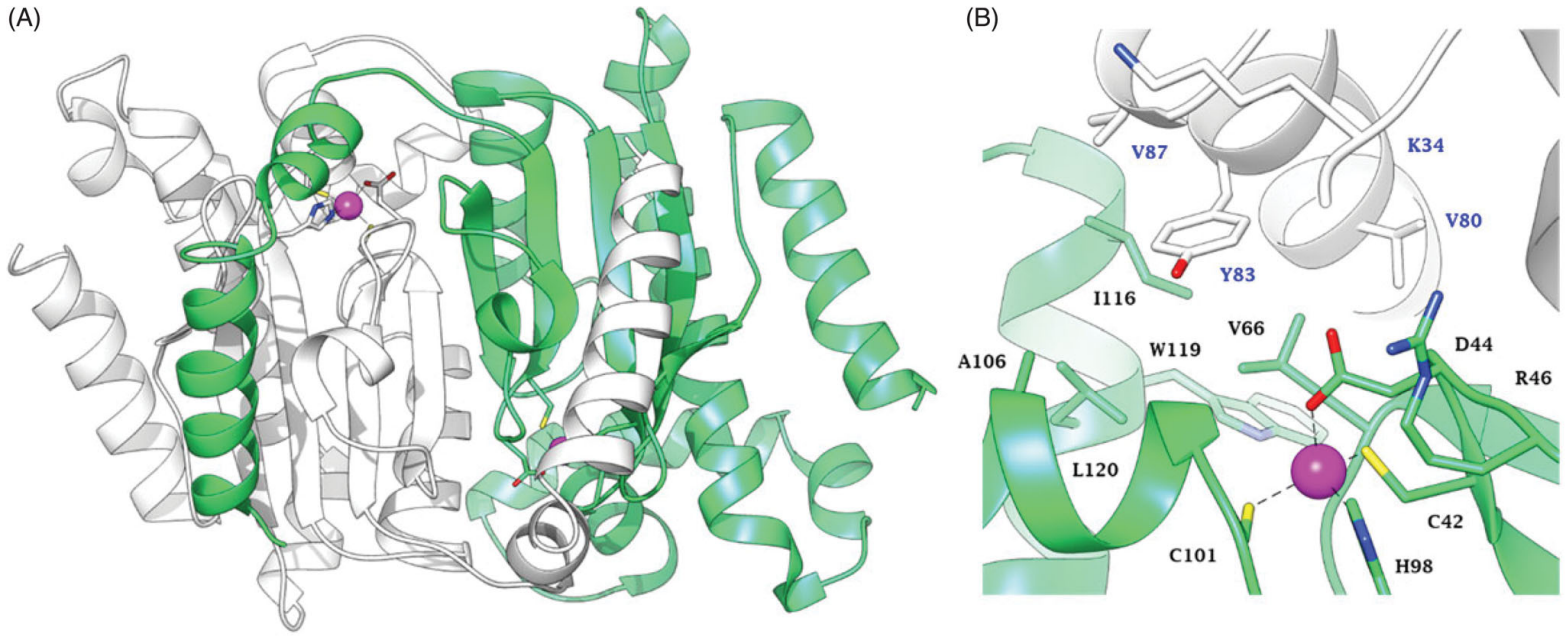
Structure of EcoCAβ (PDB code 1I6O^2^) (A) Ribbon representation of the dimer. (B) View of the active site. The Zn(II) ion is represented as a magenta sphere. Chain A and chain B are coloured white and green, respectively. Amino acids in the active site are labelled with one letter symbols (blue for chain A and black for chain B): A, Ala; C, Cys; D, Asp; H, His; I, Ile; K, Lys; L, Leu; R, Arg; V, Val; W, Trp; Y, Tyr.

A mentioned in the introduction, the crystal structure of EcoCAβ was reported in 2001^2^ (Figure 2), although no kinetic characterisation of the enzyme was reported.

As many other β-CAs have been characterised to date by X-ray crystallography^41^, EcoCAβ is a tetrameric enzyme, more precisely a dimer of homodimers. The dimer which constitutes the fundamental element for the structure and functioning of this enzyme is shown in Figure 2(A), with the catalytic Zn(II) ion situated at the bottom of a rather long and narrow active site^2^. However, when the detailed coordination geometry of the active site was inspected (Figure 2(B)), a rather surprising situation emerged: the metal ion was observed to be coordinated by four amino acid residues (Cys42, His98, Cys101 and Asp44), with no water molecule coordinated to the Zn^2+^ to form the zinc-hydroxide nucleophile in the CO_2_ hydration reaction. Thus, for a rather long period of time the catalytic mechanism of this type of β-CAs (thereafter denominated “closed active site” or “type II β-CAs”) was poorly understood, with various hypothesises being proposed, including that a water molecule acts as the fifth zinc ligand (but was unobserved in the crystal structures), which may eventually be used to form the nucleophile^2^^,^^42^. This mystery has been resolved in a very elegant study by Covarrubias et al.^43^, who used a β-CA from *Mycobacterium tuberculosis*, which was crystallised at various pH values. For pH values >8.3, the Zn(II) ion was coordinated by a His residue, two Cys residues and a water molecule/hydroxide ion, whereas at pH values <8, the coordination was as the one shown in Figure 2(B), with an Asp replacing the water molecule^43^. The active site at higher pH was thus entitled the “open” and that at lower pH was considered ‘closed’^43^. Furthermore, the mechanism by which the closed active site is opened has also been elucidated: a conserved catalytic dyad comprising an Asp and an Arg residue (Asp44–Arg46 in Figure 2(b)) is present in all β-CAs. In the closed active site enzymes, the Asp of the dyad is coordinated to the Zn(II) ion at pH values <8. At higher pH values, the carboxylate moiety of this Asp residue is involved in a strong ionic interaction with the guanidinium moiety of the Arg from the dyad and thus, liberating the coordination position around the zinc ion to allow coordination of a water molecule; that is, the key zinc-hydroxide nucleophile can be formed by the enzyme during catalysis from the open but not the closed active site^43^. For this reason, the type II β-CAs are usually catalytically active only at pH values >8.

Thus, we measured the catalytic activity of EcoCAβ at a pH of 8.3 and determined its kinetic constants (*k*_cat_ and *K*_M_) for comparison to those of the thoroughly studied human (h) CA isoforms hCA I and II, belonging to the α-CA class (Table 1) as well as a recently investigated pathogenic, fungal β-CA, from *Malassezia restrica*, MreCA^44^. The experiments were also performed in the presence of 10 µM L-Trp as an activator (Table 1) or in the presence of a sulphonamide inhibitor (data not shown).

**Table 1.** Activation of human carbonic anhydrase (hCA) isozymes I, II, MreCA and EcoCAβ with L-Trp, at 25 °C, for the CO_2_ hydration reaction^25^.

Isozyme	*k*_cat_	*K*_M_	(*k*_cat_)_L-Trp_**	*K*_A_*** (µM)
	(s^−1^)	(mM)	(s^−1^)	L-Trp
hCA I^a^	2.0 × 10^5^	4.0	3.4 × 10^5^	44.0
hCA II^a^	1.4 × 10^6^	9.3	4.9 × 10^6^	27.0
MreCA^b^	1.06 × 10^6^	9.9	9.6 × 6^6^	0.32
EcoCAβ	5.3 × 10^5^	12.9	1.8 . 10^6^	18.3

*Observed catalytic rate without activator. *K*_M_ values in the presence and the absence of activators were the same for the various CAs (data not shown). ** Observed catalytic rate in the presence of 10 µM activator. ***The activation constant (*K*_A_) for each enzyme was obtained by fitting the observed catalytic enhancements as a function of the activator concentration^41^. Mean from at least three determinations by a stopped-flow, CO_2_ hydrase method^25^. Standard errors were in the range of 5–10% of the reported values (data not shown).

^a^Human recombinant isozymes, from Ref.^8^; ^b^Fungal recombinant enzyme, from Ref.^44^, ^c^This work.

The catalytic activity of EcoCAβ is substantial for the hydration of CO_2_ to bicarbonate, with a kinetic constant *k*_cat_ of 5.3 × 10^5^s^−1^ and a Michaelis–Menten constant *K*_M_ of 12.9 mM. These kinetic parameters are in fact comparable to those of other α- or β-CAs (Table 1). In fact, EcoCAβ has an activity comparable to the human isoform hCA I. The bacterial enzyme has a *k*_cat_/*K*_m_ of 4.10 × 10^7^ M^−1^ s^−1^, whereas hCA I has nearly the same ratio (5.0 × 10^7^ M^−1^ s^−1^), indicating that the two enzymes have moderate activity overall. Acteazolamide, a sulphonamide standard CAI, inhibited this catalytic activity with a K_I_ of 227 nM (hCA I is inhibited by this compound with a K_I_ of 250 nM^6^).

The data in Table 1 also indicates that the presence of L-Trp as an activator does not change the K_M_ for either of the two enzymes belonging to the α-class (hCA I/II) as well as for MreCA and EcoCAβ, a situation also observed for all CA classes for which CA activators have been investigated so far^8^^,^^31–38^. In fact, as proven by kinetic and crystallographic data^3^^,^^20^, the activator binds in a different region of the active site than the site of substrate binding. Thus, the activator does not influence *K*_M_ but has an effect only on *k*_cat_. Indeed, a 10 µM concentration of L-Trp leads to a 3.4-fold enhancement of the kinetic constant of EcoCAβ compared to the same parameter in the absence of the activator (Table 1). For hCA I and II, the enhancement of the kinetic constant in the presence of L-Trp was rather modest, as these enzymes have a weaker affinity for this activator (Table 1). On the other hand, L-Trp has a low micromolar affinity for EcoCAβ which explains its more effective activating effect on this enzyme.

Thus, we proceeded with the investigation of activators **1**–**24** (Figure 1) belonging to the amino acid and amine chemotypes for understanding their ability to activate EcoCAβ. In Table 2, the activation constants of these compounds against the target enzyme EcoCAβ as well as hCA II and II (α-CA enzymes) and MreCA (a fungal β-CA) are shown. The following structure-activity relationship (SAR) was observed for the activation of EcoCAβ:

**Table 2.** Activation constants of hCA I, hCA II and the fungal enzyme MreCA from *M. resticta* and EcoCAβ (*E. coli*) with amino acids and amines **1**–**24**, by a stopped-flow CO_2_ hydrase assay^25^.

No.	Compound	*K*_A_ (µM)*			
		hCA I^a^	hCA II^a^	MreCA^b^	EcoCAβ^c^
**1**	L-His	0.03	10.9	12.8	36.0
**2**	D-His	0.09	43	1.84	23.7
**3**	L-Phe	0.07	0.013	2.69	12.0
**4**	D-Phe	86	0.035	0.76	15.4
**5**	L-DOPA	3.1	11.4	0.87	10.7
**6**	D-DOPA	4.9	7.8	0.70	3.14
**7**	L-Trp	44	27	0.32	18.3
**8**	D-Trp	41	12	0.89	11.5
**9**	L-Tyr	0.02	0.011	4.15	9.86
**10**	D-Tyr	0.04	0.013	7.83	17.9
**11**	4-H_2_N-L-Phe	0.24	0.15	0.61	7.34
**12**	Histamine	2.1	125	0.90	18.5
**13**	Dopamine	13.5	9.2	2.71	11.3
**14**	Serotonin	45	50	0.82	2.76
**15**	2-Pyridyl-methylamine	26	34	0.34	48.7
**16**	2–(2-Aminoethyl)pyridine	13	15	2.13	17.2
**17**	1–(2-Aminoethyl)-piperazine	7.4	2.3	0.25	14.1
**18**	4–(2-Aminoethyl)-morpholine 0.14	0.19	0.33	17.4	
**19**	L-Adrenaline	0.09	96.0	0.015	9.15
**20**	L-Asn	11.3	>100	0.93	49.5
**21**	L-Asp	5.20	>100	4.04	18.9
**22**	L-Glu	6.43	>100	5.26	18.0
**23**	D-Glu	10.7	>100	4.70	11.4
**24**	L-Gln	>100	>50	0.90	49.2

*Mean from three determinations by a stopped flow, CO_2_ hydrase method^25^. Standard errors were in the range of 5–10% of the reported values (data not shown).

^a^Human recombinant isozymes, from Ref.^8^; ^b^Fungal recombinant enzyme, Ref^44^; ^c^Bacterial recombinant enzyme, this work.

(i) A rather weak activation was observed with L-His, 2-pyridyl-methylamine **15**, L-Asn and L-Gln, which had activation constants K_A_s from 36.0 to 49.5 µM.

(ii) Medium potency activating effects were observed for the following derivatives: D-His, L- and D-Phe, L- and D-Trp, D-Tyr, histamine, dopamine, 2-(aminoethyl)pyridine/piperazine/morpholine (compounds **16–18**), L-Asp, L- and D-Glu. These derivatives have K_A_s from 11.3 to 23.7 µM (Table 2). These activators belong to the heterogeneous classes of amines and amino acids, with both aromatic, heterocyclic and aliphatic representatives in both series. Thus, the SAR is rather challenging to delineate definitively.

(iii) The most effective EcoCAβ activators were L- and D-DOPA, L-Tyr, 4-amino-Phe **11**, serotonin and L-adrenaline, which had *K*_A_s from 2.76 to 10.7 µM. The most effective compounds of this subseries were serotonin (*K*_A_ of 2.76 µM) and D-DOPA (*K*_A_ of 3.14 µM). In addition, for the enantiomers of this amino acid, the D-enantiomer was 3.4-times more effective as an activator than the L-enantiomer.

The mechanism of action of these CAAs probably involves the facilitation of the rate-determining step of the catalytic cycle [Equation (3)], as for the α-CAs^8^. However, no X-ray crystal structures of the activator-enzyme complexes have been characterised so far for the β-CAs. The proton shuttling residue for this class of enzymes is yet to be definitively established, but as shown in Figure 2(A), the long channel which constitutes the active site of β-CAs would easily accommodate such small molecules as the amines and amino acids investigated here, providing in this way a more efficient proton shuttling between the zinc-coordinated water molecule and the aqueous environment.

## 4. Conclusions

The β-CA from the widespread bacterium *Escherichia coli* EcoCAβ has been investigated here for the first time for its catalytic properties as well as for enzymatic activation by a panel of amino acids and amines. EcoCAβ has substantial catalytic activity for the hydration of CO_2_ to bicarbonate, with a kinetic constant *k*_cat_ of 5.3 × 10^5^ s^−1^ and a Michaelis–Menten constant *K*_M_ of 12.9 mM. The most effective EcoCAβ activators were L- and D-DOPA, L-Tyr, 4-amino-Phe, serotonin and L-adrenaline (K_A_s from 2.76 to 10.7 µM). Weak activation was observed for L-His, 2-pyridyl-methylamine, L-Asn and L-Gln, with activation constants *K*_A_s in the range of 36.0–49.5 µM. D-His, L- and D-Phe, L- and D-Trp, D-Tyr, histamine, dopamine, 2-(aminoethyl)pyridine/piperazine/morpholine, L-Asp, L- and D-Glu showed *K*_A_s in the range of 11.3–23.7 µM. CA activators may play a role in bacterial virulence and colonisation of the host, although no detailed studies in this area are available to date.
